# What’s knowledge is prologue: celebrating the 40th anniversary of the p53 discovery

**DOI:** 10.1093/jmcb/mjz064

**Published:** 2019-08-22

**Affiliations:** Institute of Biochemistry and Cell Biology, Shanghai Institutes for Biological Sciences, Chinese Academy of Sciences, Shanghai 200031, China E-mail: wujr@sibs.ac.cn

***I***t is my great honor to deliver a welcome speech as the Editor-in-Chief of *Journal of Molecular Cell Biology* (JMCB) and the Chair of *JMCB Symposium 2019: The Legend of p53 vs. Cancer*. Please allow me to borrow the logion ‘what’s past is prologue’ from the great English playwright William Shakespeare as follows:

What’s knowledge is prologue. This phrase could be applied for the most famous p53. Since the *TP53* gene was identified by Dr Levine and others (DeLeo et al., 1979; Kress et al., 1979; Lane and Crawford, 1979; Linzer and Levine, 1979), nearly a hundred thousand of papers about p53 so far have been published. At the early stage of p53 studies, researchers paid attention to its function in transcriptional regulation; later, researchers uncovered various non-transcription functions (Lu, 2017; Zhou et al., 2017). p53 not only functions in the physiological and pathological processes (El-Dahr et al., 2017; Tackmann and Zhang, 2017), but is also involved in the factitious biological processes such as generating iPS cells or gene editing by CRISPR/Cas9. Like the Swiss Army knife, p53 could play various roles during different biological processes.

What’s knowledge is prologue. This phrase could be applied for the most complicated p53. In the beginning, the *TP53* gene was regarded as an oncogene, but soon re-defined as a tumor suppressor. The *TP53* gene has the highest mutation rate among genes identified in the human genome; previous work showed that various mutations of the *TP53* gene existing in around 50% of tumor types result in the loss of its tumor suppressive function. However, a recent study reported that one particular mutation in the *TP53* gene could generate super-tumor suppression (Mello et al., 2017). In addition, p53 could interact with different partners and then play opposite roles, as either tumor suppressor or oncogene. Just at the beginning of this year, one work uncovered the dark side of p53: wild-type p53 can promote the progression of liver cancer (Kim et al., 2019). The studies on p53 clearly demonstrate the complexity and randomicity of living systems.

What’s knowledge is prologue. This phrase would not be limited to the research of p53. The knowledge as ‘islands’ locates in the unlimited ‘sea’ of the unknown. The more increasing the knowledge is, the more growing the related unknown area is. It is the challenge of the research, and it is the delight of the research!

*[Opening remarks at JMCB Symposium 2019, Hangzhou, China. May 10, 2019.]*
